# Ecohydrological threats to *Colophospermum mopane* in southern Zimbabwe

**DOI:** 10.4102/jamba.v11i2.714

**Published:** 2019-07-02

**Authors:** Tendayi Gondo, Agnes Musyoki, Aina T. Adeboyejo

**Affiliations:** 1 Department of Urban and Regional Planning, School of Environmental Sciences, University of Venda, Thohoyandou, South Africa; 2 Department of Geography and Geo-Information Sciences, School of Environmental Sciences, University of Venda, Thohoyandou, South Africa; 3 Department of Urban and Regional Planning, Ladoke Akintola University of Technology, Ogbomoso, Nigeria

**Keywords:** Ecohydrology, *Colophospermum mopane*, Ecosystem Services, Threat Assessment, Dryland

## Abstract

Rapid ecohydrological changes in semi-arid landscapes are increasingly threatening humanity’s life-support systems and eroding many of the ecosystem services (ESs) upon which humans occupying such regions depend. Knowing which services and ecohydrological changes to be most concerned about is indispensable to maintaining the general health of such ecosystems and for developing effective ecosystem management practices. In the semi-arid regions of southwestern Zimbabwe where a large population of rural households depend on ESs extracted from the *Colophospermum mopane* tree, such understanding may be critical in reversing potential ES losses that may have catastrophic effects on the lives of many. We surveyed a total of 127 rural households who occupy the semi-arid landscapes of the *Colophospermum mopane* belt in southern Zimbabwe. We assessed the ecohydrological conditions characterising ecosystems where they obtain ES provisioning goods using a number of ecohydrological variables commonly cited in the literature on ecohydrology. Building on principal component analysis (PCA), we employed a hierarchical agglomerative clustering method to create unique clusters of households that depicted different levels of risks or threats associated with their ES provisioning harvesting practices. Multiple regression analysis was further performed to identify significant ecohydrological cluster-defining variables. Our results showed that spatial differences in ecohydrological parameters resulted in four distinct ES resource thresholds depicting four categories of risks that households face in extracting such resources in nearby landscapes. We concluded by proposing a number of landscape restoration or management practices targeted at reversing potential ES losses and subsequently safeguarding the livelihoods of many who depend on ESs.

## Introduction

The need to understand the scale and urgency of threats to ecosystem services (ESs) is crucial, as such a concern informs the development of guide plans targeted at averting and alleviating these threats. Evidence of such a concern is widespread. The Millennium Ecosystem Assessment (2005), for example, established an understanding of ESs and how human activities posed a threat to them (Cardinale 2012). The assessment concluded that human-altered ecohydrological processes were responsible for the degradation of 60% of ESs or the subsequent unsustainable use thereof (Costanza 2014). Ecohydrological threats to ESs and other related concerns have also seen the Intergovernmental Platform on Biodiversity and Ecosystem Services (established in 2012) emphasising the need to synthesise scientific evidence on the state of biodiversity and ESs and the need to provide policy-relevant knowledge for decision-making purposes (Díaz 2015). Fear of extinction of some species and the general collapse of some ecosystems inspired the International Union for Conservation of Nature (IUCN) to generate a ‘Red List’ classification system (Maron et al. 2017). Such a classification system sought to provide an informed understanding of the scale and urgency of threats to species and ecosystems and guide plans to avert and alleviate these threats. The Red List classification of threatened ecosystems, in principle, requires researchers and policy-makers alike to classify ecosystems using a number of categories, including Data Deficient, of Least Concern, Near Threatened, Vulnerable, Endangered, Critically Endangered, and Collapsed. In this analysis, we contend that such a classification system is a step closer to developing a framework that is necessary for creating an obvious link between the science of ecosystem assessment and the policy imperative to safeguard ES provision.

Despite significant advances in the understanding of ecohydrology and ES provisioning across science and policy arenas, valuation of ecohydrological threats to ESs to guide sustainable conservation practices remains challenging. In the semi-arid regions of southwestern Zimbabwe where a large population of rural households depends on ESs extracted from the *Colophospermum mopane* tree, such a challenge is further compounded by data scarcity and the lack of an appropriate framework that will permit such valuations. Three main areas of concern motivated this analysis. These include:

- understanding the degree to which the adequate and sustainable provision of given ESs is threatened
- understanding the type of risks confronting resource users as a result of potential losses in the provision of ESs
- understanding the critical ecohydrological parameters threatening loss of such ESs.

Following this introduction, we discuss the need to assess threats in ESs. We proceed to review extant literature that helped in developing the conceptual framework of the study. A methodology section that outlines the research instruments used is then discussed. Results are then presented and discussed before the final conclusions are drawn.

### The need to assess threats to ecosystem services

As most ecohydrological processes have been found to have a direct influence on the quality and quantity of ESs, a number of models designed to perform such assessments have been developed. Some ecohydrological models have, for instance, developed probabilistic model frameworks to predict the impact of climate and soil type variations on conditions of water stress of vegetation (Porporato et al. 2002; Rodriguez-Iturbe et al. 2001). Other hydrological models have also tried to predict the hydrological consequences on ESs. The vegetation dynamic model has been used to predict the hydrological consequences on ES variables. Such studies have predicted that ES variables are significantly influenced by what Han et al. (2015) have referred to as ‘environment variables’ such as soil water content and groundwater depth.

Despite the bulk of such assessments, very few studies have attempted to explicitly incorporate the element of risk or threat to ESs. Understanding the risk of extinction of certain ESs or collapse of ecosystem attributes will certainly require approaches that go beyond traditional assessment practices that seek to quantify only the amount of ESs available in a particular landscape (Maron et al. 2017). According to Maron et al. (2017), assessment efforts need to be directed at developing a standard set of criteria for pinpointing when and to what degree adequate provision of an ES in a given landscape is at risk and shedding light on how immediate the risk of complete loss of the service is. Absence of such a standardised set of criteria has seen us lacking a consistent basis for prioritising management interventions in overcoming threats to ESs or at least supporting their recovery.

### Categorising ecohydrological threats to ecosystem services: The assessment framework

The need to categorise threats to ESs has been underscored in the preceding literature. How to categorise is a daunting task as we currently lack an assessment framework that will help pinpoint when and to what degree adequate provision of ESs in a given environment is at risk, or how immediate the risk of complete loss of the service is (Maron et al. 2017). A plausible entry point to developing one is to define what constitutes ‘threat’. Within the context of ESs, threat should be conceptualised in two main ways. Firstly, we make reference to what we refer to as a ‘loss perspective’ and define threat as loss of service provision to a group of beneficiaries situated in a defined geographical location that is characterised by certain loss bearing ecohydrological characteristics. Such a perspective allows us to go beyond the traditional conceptualisation of threat that defines threat as a global loss of an ES. A second perspective that has been characterised in other literatures (see Maron et al. 2017) as a ‘supply versus demand’ perspective requires us to define threat as a failure of the existing supply mechanisms to meet the demand. In other words, ESs beneficiaries suffer in cases where the existing ecohydrological conditions are not sufficient enough to meet existing demands. Assessing threats to ESs therefore requires a framework that incorporates a human dimension – particularly by looking at the consequences of ecohydrological conditions characterising a particular landscape to benefits that accrue to humans.

**FIGURE 1:**
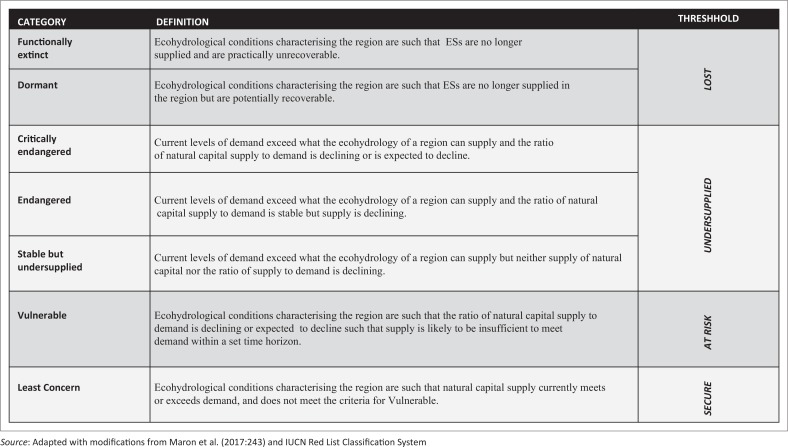
A framework to categorise threats to ecosystem services.

We argue that net gains to humans are maximised when the supply of natural capital is able to meet the demand for that service by people. Maximising net gains would also mean that the resulting ecohydrological processes are able to ensure a steady supply of ESs for human consumption. Where such conditions are not permitting and where supply conditions of an ecosystem are not guaranteeing that demand of ESs will be met, risks to the well-being of people occur. Taking the cue from the risk register approach proposed by Mace (2015) and the Red List proposed by IUCN as well as the threat assessment approach developed by Maron et al. (2017), we developed a threat assessment framework (Figure 1).

### Dryland ecohydrology threats to ecosystem services: A review of critical issues

Drylands are a critical terrestrial system of the Earth that is characterised by low water availability (Pravalie 2016). The quality and quantity of ESs available to communities occupying dryland or semi-arid landscapes are shaped by the hydrology of such landscapes. Like in many other ecosystems, semi-arid and arid landscapes are water controlled in that water availability is the single most important driver of the structure and organisation of (water related) ESs (Lehmann et al. 2014; D’Onofrio et al. 2015). The state of ecohydrological systems of arid and semi-arid landscapes has often been understood within the context of environmental variables such as conditions of water stress for vegetation (Porporato et al. 2002; Rodriguez-Iturbe et al. 2001), soil water content and groundwater depth (Shi et al. 2012; Zhao et al. 2005).

Certainly understanding dryland ecosystem responses to temporal and spatial changes in ecohydrological conditions is critical, as these ecosystems cover nearly 40% of the global land surface (Buntinga, Munsona & Villarrealba 2017). Such ecosystems also play an important role in supporting development for large human populations and providing ESs (Buntinga et al. 2017). A number of critical ecohydrology parameters and the important connections that exist between them and the subsequent effect they have on the availability of ESs have been extensively reviewed.

Commonly discussed ESs impacting parameters include, among others:

- drainage density (Tooth 2000)
- flood and spatial flow variations (Kelly & Olsen 1993 in Tooth 2000)
- precipitation and steam order characteristics (Strahler 1957; Lehmann et al. 2014; D’Onofrio et al. 2015)
- vegetation characteristics (Buntinga et al. 2017; House et al. 2003; Li et al. 2014; Sala et al. 2012; Xu et al. 2015)
- Soil moisture (Van Wie, Adama & Ullman 2013)
- land degradation (Chasek et al. 2015; Grainger 2015; UNCCD 2012, 2013)
- topography (Davies et al. 2014; Levick et al. 2010; Muvengwi et al. 2016).

The hypothesised relationship between such variables and ESs provisioning is presented in the methodology section.

## Materials and methods

**FIGURE 2:**
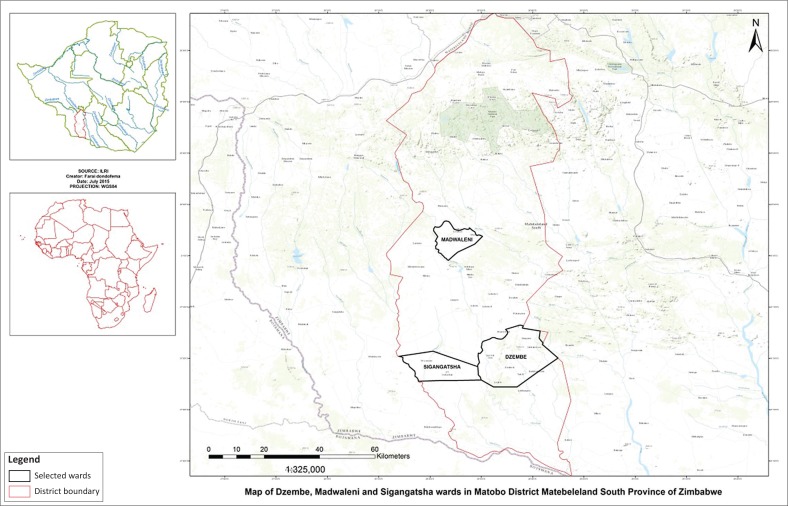
Selected study sites in Matobo District, Zimbabwe.

The study hypothesised that mesoscale variability in ecohydrology parameters has a direct and significant influence on the availability of ESs to households. It also hypothesised that ecohydrological threats to the availability of ESs can be categorised in such a manner that specific resource use thresholds can be discerned. The study was carried out in a sample of three wards in Matobo District of Zimbabwe (Figure 2).

We used the ward as a mesoscale sampling unit of analysis instead of the village as we envisaged that places where ES provision was sourced had overlapping tendencies at village level that would have complicated the analysis. The choice of the wards was guided by variations in vegetation cover with Sigangatsha ward characterised by evenly distributed vegetation, and the other two wards were characterised by sparse and uneven vegetation. Madwaleni ward was differentiated from Dzembe ward with the level of degradation as the ward is closer to major urban centres. A total of 127 households were randomly selected in these three wards for questionnaire administration. Forty-four households (35%) were selected from Sigangatsha ward, 47 (37%) from Madwaleni ward and 36 (28%) from Dzembe ward. The main survey was conducted in 2014–2015 season, although a series of other surveys that have complemented this analysis have been carried out since 2002.

### Ecosystem service value assessment

Ecosystems in semi-arid landscapes provide production, regulation, support and cultural entertainment service functions, and these functions in turn create a series of natural environmental conditions and socio-economic benefits for human survival and development (De Groot et al. 2002). Measuring such benefits, particularly the indirect ones, is a big challenge. In this study, we focused on benefits associated with ESs provision. We used focal ESs such as provision of food, raw materials and medicinal resources – similar to those used in the study by Ramirez-Gomez et al. (2015). We identified nine provisioning services that were deemed important by the local people through a series of participatory group discussions. The provisioning services that were considered in this analysis and that are also consistent with what *Colophospermum mopane* landscape can offer to rural households are supply of timber (for construction of houses and livestock kraals), thatch (grass for roofs), resins (tree exudate used as glue or sealant), wild fruits, bush meat (animals hunted for meat) – including mopane worms, fish (caught for commercialisation), natural medicines, materials for making crafts and traditional tools (fibres, stems and leaves) and ornamental resources (fibres, trees and tree bark used for making clothes for traditional dances and celebrations).

In developing the ES constructs, we employed the concept of a service provisioning area (SPA) referring to the source of ESs (Syrbe & Walz 2012). We only recorded the extent of household involvement in the collection of nine provisioning ESs within the confines of their village. Resource extraction behaviours of households were used as a proxy measure of ES values – largely depicting the amount of ESs derived from a given ecohydrological landscape using the following formula:

$${\text{ESV}}_{\text{ij}}=e_{\text{ij}}$$ [Eqn 1]

where ESV_**ij**_ is the value attached to ***j***th ES obtained by the household from ecosystem *i*. *e*_ij_ is the amount of the *j*th ES obtained by the household from ecosystem *i*. The ESV_**ij**_ value was computed for the wet resource state (WRS) and dry resource state (DRS) as explained below.

### Accounting for spatial and temporal variations

Although our study focused more on spatial changes, we could not overlook temporal variation. We attempted to incorporate the temporal dimension, by employing a proxy measure that we found to be ideal in depicting the influence of variations in precipitation on availability of ESs. We borrowed insights from ‘pulsing hydrology’. Pulsing hydrology informs us that the spatial and temporal dynamics of an ecosystem are often influenced by resource availability and timing (Muvengwi et al. 2016). As argued by Parsons and Thoms (2013), there is a need to examine ESs associated with vegetation trends in flood, rain and dry states. Related studies have shown that vegetation and associated ESs increase as one moves from a dry to a flood state (Thapa, Thoms & Parsons 2016). To capture such temporal shifts, Thapa et al. (2016) distinguished between two important states in their research on semi-arid topic. These include the DRS (essentially the dry state) and the WRS (essentially the rain and flood state). The researchers therefore expected the level of resource extraction by communities to be relatively high in the WRS than in the DRS.

### Variable determination and measurement

A number of ecohydrology parameters that would pose a threat to the supply of ESs to rural households were identified based on a critical review of the literature. The variable selection process was based not only on relationships that were clearly apparent on a variety of data sets but also on an ecological vulnerability assessment indicator system that we constructed. Following examples from Zang et al. (2017), we constructed an ecological vulnerability assessment indicator system using the ‘exposure–climate sensitivity–adaptive capacity’ framework according to the theory of ecological vulnerability (Beroya-Eitner 2016 in Zang et al. 2017). Our threat assessment approach was not misplaced, as empirical evidence from elsewhere has shown that ecological vulnerability is often assessed by combining the characteristics of study subjects and the objectives of the study based on the ‘exposure–sensitivity–adaptability’ framework (Zang et al. 2017). The final selection of ecohydrological variables was however severely constrained by data scarcity. We did not view this as a methodological deficiency as some studies have acknowledged that dryland regions are poorly gauged and therefore lack a detailed understanding of their ecohydrology (Jarihani et al. 2015). Variables that were selected for final analysis, their measurement metrics and hypothesised relationships are summarised in Table 1.

**TABLE 1:** Threat assessment indicators system used.

Criterion layer	Indicator layer	Measurement metric	Description hypothesised relationships
Exposure indicator	Topography/Catena influences	Based on a 4-point Likert scale used in a study by Muvengwi et al. (2016): 4 = upper; 3 = middle; 2 = bottom; 1 = floor 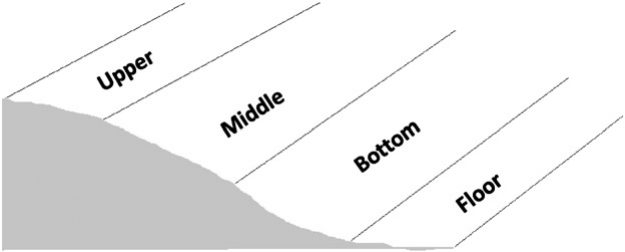	Based on individual transect walks in places where ES resource extraction takes place. The lower the catena, the greater the environmental stress on ESs (Davies et al. 2014; Levick et al. 2010; Muvengwi et al. 2016).
	Drainage density (extent of channelisation)	A 3-point Likert scale was used as follows: 3 = high (higher than the combined drainage density average for all wards) 2 = average 1 = low (higher than the combined drainage density average for all wards)	The greater the extent of landscape channelisation, the greater the environmental stress on ESs (Tooth 2000).
Climate sensitivity indicators	Flood water characteristics	A 2-point Likert scale was used as follows: 2 = upstream 1 = downstream	The lower the level of stream (i.e. whether down-stream versus upstream) the greater the environmental stress on ESs. This is because semi-arid river floods are always subject to downstream volume decreases due to transmission losses resulting from infiltration of floodwaters. Further losses result from overbank flooding and evaporation of flood waters (D’Onofrio et al. 2015; Lehmann et al. 2014).
	Stream order characteristics	Based on stream order classification method by Strahler (1957)	The lower the stream-order the greater the environmental stress on ESs as less water associated with lower stream orders is insufficient to sustain many ESs. (D’Onofrio et al. 2015; Lehmann et al. 2014).
	Soil particle size	A 5-point classification system: 5 = coarse sand; 4 = sand; 3 = fine sand; 2 = very fine sand; 1 = silt and Clay 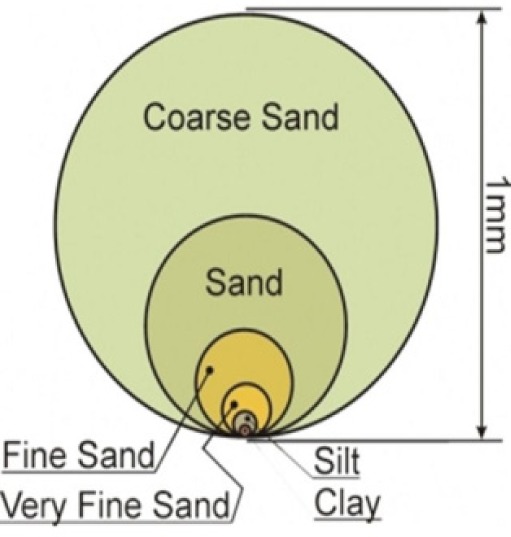	Moderate indicator: excess soil, sand or clay content is unfavourable for plant growth and many of the ESs associated with plants (Van Wie et al. 2013)
Adaptive capacity indicators	Soil moisture storage abilities	3 = good – better insulation (mostly vegetated, evidence of shrubs, crop residue) 2 = fair – fairly insulated (both extreme scenarios co-existing) 1 = poor – poor insulation (evidence of bare soils, herbaceous vegetation, highly degraded landscapes)	The availability of soil moisture controls plant processes such as transpiration, primary production and nutrient uptake simultaneously. The lower the soil moisture storage abilities, the greater the environmental stress on ESs. This is because water- and vegetation-related ESs in semi-arid landscapes requires sufficient water storage in the soil profile to ensure adequate water is available for plant growth as the majority of annual precipitation occurs during the non-growing season (Van Wie et al. 2013).
	Type of plant communities	3 = mostly woody (i.e. trees and shrubs) 2 = partly woody and partly herbaceous vegetation 1 = mostly herbaceous (mainly grasses and herbs)	Compared with woody vegetation, herbaceous vegetation is more sensitive to precipitation events and water stress and displays higher turnover rates. Woody vegetation on the other hand has been found capable of maintaining growth in drier soils than herbaceous vegetation. Woody vegetation loses biomass more slowly when the soil is drier than herbaceous vegetation. Woody plants also cope better than herbaceous plants with wind erosion, sand burial and grazing. Slow-growing woody vegetation requires fewer soil nutrients than fast-growing grasses (Buntinga et al. 2017; House et al. 2003; Li et al. 2014; Sala et al. 2012; Xu et al. 2015).
	Land degradation	3 = land under non-degrading use 2 = land under degrading use 1 = degraded Observable land use practices (e.g. prevalence of stumps owing to tree cutting) allow us to identify lands under ‘non-degrading use’ requiring sustaining, lands under ‘degrading use’ and therefore needing mitigation and those that are already degraded requiring restoration measures.	Degradation or near degradation scenarios in semi-arid environments exhibit different ecohydrological conditions that limit the amount and quality of ESs available for use. Negative indicator: In areas with a sparse or uneven vegetation cover, we expect less moisture to be available for ESs since rainfall often occurs at high intensities and is subsequently associated with high runoff coefficients (Chasek et al. 2015; Grainger 2015; UNCCD 2012, 2013).

ESs, ecosystem services.

### Statistical procedures

Prior to resolving the indicators, raw data were processed for ‘homogenisation’ and ‘non-dimensionality’, which is a standard requirement as there can then be questions of examining the homogeneity across the sites of the distribution of the scaled values (Hall 2003). Study constructs were first tested for normality. Ecohydrological indicator variables were further explored for reliability and validity through Exploratory Factor Analysis (EFA) using the Principal Component Analysis (PCA) method with varimax rotation. Important measures of reliability and validity were computed, including Cronbach’s alpha, composite reliability and the Average Variance Extracted (AVE). Both statistical measures sought to estimate internal consistency associated with the scores derived from the data scales (Hair et al. 2006). To evaluate discriminant validity, the AVE was calculated (Voorhees et al. 2015). The AVE was calculated as follows:

$$AVE=\frac{\sum \lambda_{i}^{2}}{\sum \lambda_{i}^{2}+\sum_{i}\text{var}(\varepsilon_{i})}$$ [Eqn 2]

where λ is the factor loading of item *i* and var(ε) is the variance of the error of item.

Within the EFA framework, the Kaiser–Meyer–Olkin (KMO) statistics and Bartlett’s test of *sphericity* were performed to examine the data suitability for PCA.

### Hierarchical agglomerative cluster analysis

Most ecohydrological studies that seek to draw patterns from large data sets usually employ some form of clustering (Canedo-Arguelles et al. 2016; Chang, Han & Zhong 2009). Cluster analysis (CA) is a group of multivariate techniques whose primary purpose is to assemble objects based on their common characteristics (Kim et al. 2007). Central to all CA studies, hierarchical agglomerative clustering is the most common approach that is used to generate intuitive similarity relationships between any given data sets.

Hierarchical agglomerative CA was performed on the normalised data set. Conceptually, the analysis denotes ecohydrological threat outcomes associated with variable *i* in ecosystem and landscape *j* as *Y*^ij^. This outcome is represented in Equation 1 as a function of the individual ecohydrological characteristics, Xqij, and a model error, rij (Bryk & Raudenbush 1992).

$${\text{Y}}_{\text{ij}}=\beta_{0\text{j}}+\beta_{1\text{j}}{\text{X}}_{1\text{ij}}+\beta_{2\text{j}}{\text{X}}_{2\text{ij}}+\ldots +\beta_{\text{nj}}{\text{X}}_{\text{nij}}+{\text{r}}_{\text{ij}}$$ [Eqn 3]

where r_ij_~N(0,σ^2^).

Cluster analysis reduces the large number of ESs users into a small number of homogeneous groups, classified according to common ecohydrological attributes that depicted different resource thresholds in landscapes where resources are obtained. The various resource thresholds created were then used to draw important connections between associated ecohydrological conditions and the risk of loss of valuable livelihood source of the resource users.

### Regression analysis

The use of regression to estimate the influence of ecohydrological parameters is not new (Canedo-Arguelles et al. 2016; Han et al. 2015). We performed multiple regression analysis to determine which of the cluster-defining ecohydrological attributes were significantly shaping the resultant resource thresholds and their associated threats to the availability of ESs in both the WRS and the DRS. We used ES values as the dependent variable. Because of inherent normality problems associated with our dependent variables, we used the *Box-Cox Transformation formula* to stabilise the variance, make data more normal and subsequently improve the validity of measures of association depicted by the regression model.

To predict the model best fit, the analysis used the *R*^2^ *measure* and performed an ANOVA test. Collinearity was diagnosed using the tolerance and the Variance Inflation Factor (VIF) as guided by O’Brien (2007). We used linear residual plots to detect model inadequacies in regression diagnosis. Specifically, we used such plots to assess nonlinearity and heteroscedasticity in regression diagnostics. Following the cue from Weisberg (1985) and Montgomery and Peck (1992), we assumed that a null linear residual plot shows that there are no obvious defects in the model and that a curved plot indicates nonlinearity. We also concluded that a fan-shaped or double-bow pattern would indicate non-constant variance.

### Ethical considerations

In carrying out the study as well as in disseminating the research findings, the authors declare that all ethical issues in research have been addressed and that there has been no conflict of interest.

## Results and discussion

Results show that the construct developed presented overall, adequate reliability and convergent validity (Table 2). Study constructs showed a higher AVE than the square correlation, which also indicates adequate discriminant validity (Hair et al. 2006).

**TABLE 2:** Normality, reliability and validity of study constructs.

Study construct	Number of items	Normality measures		Cronbach’s alpha (≥ 0.6)	Composite reliability (≥0.6)	Average variance extracted (≥ 0.5)
		Skewness	Kurtosis			
Ecohydrology	5	0.202	−1.095	0.902	0.933	0.7345

Source: Adapted using levels of acceptance according to Hair et al. (2006)

As the independent variables are not constituted of constructs that have already been developed and validated in the literature, they were analysed by using EFA. The EFA approach was considered useful as it would allow possible renaming of study constructs to cater for variables of overlapping nature. In a first EFA, some variables showed low commonalities and were excluded. Removed variables included drainage density, stream order and soil particle size. A new EFA was performed indicating the adequacy of this analysis to explain the correlations between variables. PCA retained a latent data structure (Table 3) that can be compared to the basic elements of ecohydrology discussed in the preceding literature.

**TABLE 3:** Principal component matrix.

Eco hydrology variable	Component
Extraction method: Principal component analysis	1
Flood characteristics	834
Soil moisture storage abilities	0.905
Catena influences	0.937
Type of plant community	0.751
Land degradation	0.846

Extraction method: principal component analysis.

One component was extracted.

**FIGURE 3:**
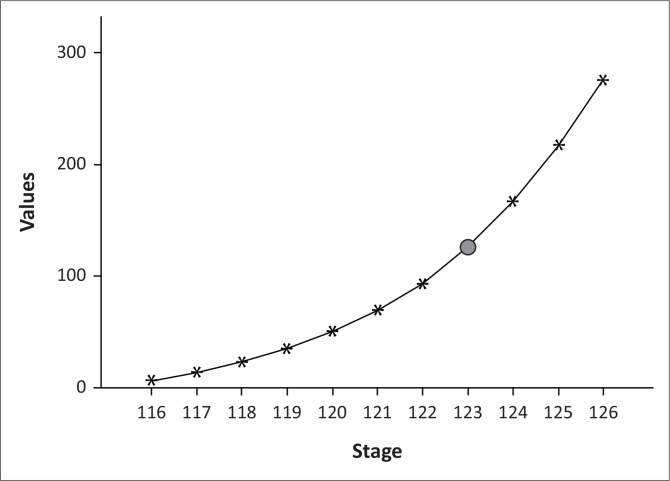
Agglomeration curve: as the ‘step of elbow’ shown by a big black dot appears to be at case number 123, a four-cluster solution should be used (i.e. 127 - 123 = 4).

A four-cluster solution was discerned through hierarchical agglomerative CA (Figure 3).

The total number of resource users in each cluster is shown in Table 4.

**TABLE 4:** Number of ecosystem service users occupying each cluster type.

Cluster type	Frequency	Per cent	Cumulative (%)	Bootstrap for per cent^a^			
				Bias	Std. error	BCa 95% confidence interval	
						Lower	Upper
1	54	42.5	42.5	0.1	4.4	34.6	51.2
2	20	15.7	58.3	0.0	3.4	10.2	21.3
3	32	25.2	83.5	−0.1	3.8	18.9	31.5
4	21	16.5	100.0	0.0	3.3	11.0	22.0
**Total**	**127**	**100.0**	**-**	**0.0**	**0.0**	**-**	**-**

BCa, bias corrected accelerated.

a , Bootstrap results are based on 1000 bootstrap samples. The bias-corrected accelerated approach was used.

A Kruskal–Wallis *H*-test showed that the cluster system created was relatively stable (χ^2^ = 126; *df* = 3; *p* < 0.05). The relative stability of such a cluster system was further reflected in the pairwise comparison of individual clusters. Results showed no serious cases of cluster overlap (Table 5).

**TABLE 5:** Pair-wise comparison of clusters based on Kruskal–Wallis test and ANOVA test results.

Pair description Cluster *a* – Cluster *b*	Test statistic (χ^2^)	Std. error	Standardised test statistic	*p*	Adjusted *p*
Cluster 1 – Cluster 2	−37.000	9.134	−4.051	0.000	0.000
Cluster 1 – Cluster 3	−63.000	7.784	−8.093	0.000	0.000
Cluster 1 – Cluster 4	−89.500	8.974	−9.974	0.000	0.000
Cluster 2 – Cluster 3	−26.000	9.946	−2.614	0.009	0.054
Cluster 2 – Cluster 4	−52.500	10.902	−4.816	0.000	0.000
Cluster 3 – Cluster 4	−26.500	9.799	−2.704	0.007	0.041

Each row tests the null hypothesis that Cluster *a* and Cluster *b* distributions are the same. Asymptotic significances (two-sided tests) are displayed. The significance level is 0.05.

In all four distinct clusters, we observed that households derive a host of ES provisions from *Colophospermum mopane* that range from construction poles, fencing posts, carvings and furniture, tools and implements, household utensils, firewood, rope, gum, medicine, leaf litter through to livestock browse and edible caterpillars (mopane worms). The greatest ecohydrological threats to ES provisioning were found to be associated with cluster type 2 and cluster type 4 households. The worst affected households are in cluster 4 where most of the ESs are sourced from landscapes where ecohydrological conditions are such that ESs are no longer supplied and are practically unrecoverable. A few instances where they can be potentially recoverable through appropriate restoration measures were however identified. Most secure ES provision-dependent livelihoods were found to be associated with cluster 1 and cluster 3 households. Of least concern are cluster type 3 households who mostly obtain ESs in landscapes where ecohydrological conditions are such that natural capital supply currently meets or exceeds demand and does not meet the criteria for vulnerable. Although the ES situation for cluster 1 households might be defined as stable, current human practices are such that ratio of natural capital supply to demand is declining or expected to decline, making such households vulnerable to future risk.

**FIGURE 4:**
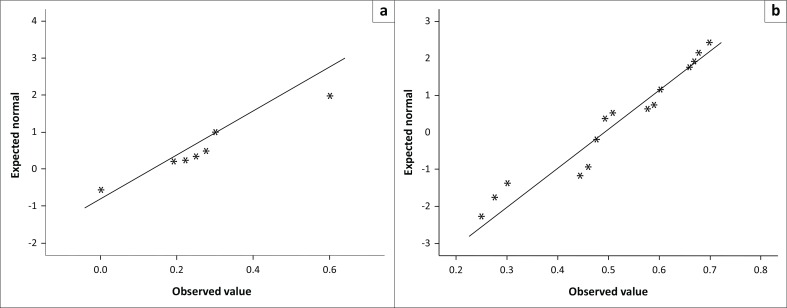
(a & b) Normalised plots for ecosystem services provision (wet resource state and dry resource state).

There was however an additional need to adopt a more robust statistical method that would indicate the significance of each cluster-defining attribute in relation to hypothesised links to ESs provisioning in both the WRS and the DRS. For this reason, multiple regression was conducted. First the dependent variable was normalised using the Box Cox Transformation formula. Normalised plots for both resource scenarios are shown in Figure 4.

The two dependent variables had no sampling adequacy issues (Field 2005) with a computed KMO statistic of 0.5. In addition, Bartlett’s test of sphericity with a chi-square value of 57.422 (*df* = 1) was highly significant at *p* < 0.001, indicating that there were adequate relationships between the ESs provisioning variables included in the analysis (Field 2005). PCA results also depicted high commonalities as evidenced by high factor loadings of 0.897 for ES provision study constructs for both the WRS and the DRS.

**FIGURE 5:**
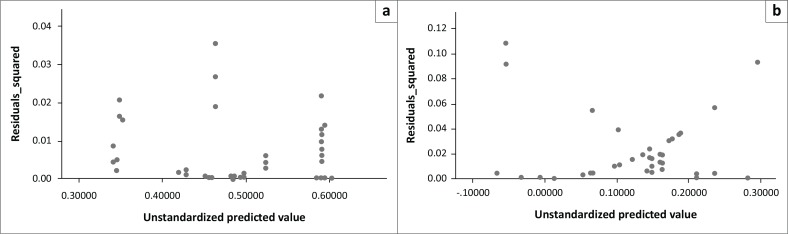
Linear residual plots (a) for wet resource state and (b) for dry resource state, indicating the presence of heteroscedasticity.

Our observed study variables fitted well on the proposed regression model as indicated by the *R*-square change and ANOVA test results. No problems of multicollinearity were detected as indicated by the collinearity diagnostics statistics in Table 6. By inspecting the computed linear residual plots, we however observed that the proposed model had a serious challenge of heteroscedasticity, which is a complete violation of the assumption of homoscedasticity associated with regression models (Figure 5). We overcame this challenge by transforming all model variables into Napier’s logarithms and testing the final outcome for heteroscedasticity using Levene’s test.

**TABLE 6:** Regression coefficients.

Season	Target layer	Independent study variable	Standardised coefficients^a,b^		Collinearity statistics		*R*^2^
			*β*	*p*	*Tolerance*	VIF	
WRS	*Demographics*	Ward	−0.054	0.390	0.856	1.168	0.603
		Household income level	−0.543	0.000	0.788	1.270	
		Flood characteristics	0.564	0.000	0.371	2.699	
	*Ecohydrology*	Soil moisture storage abilities	0.168	0.115	0.296	3.383	
		Catena influences	−0.198	0.062	0.300	3.331	
		Type of plant community	0.186	0.025	0.496	2.014	
		Land degradation	−0.255	0.005	0.426	2.350	
DRS	*Demographics*	Ward	0.122	0.151	0.537	1.861	0.389
		Household income level	−0.032	0.838	0.794	1.260	
		Flood characteristics	0.129	0.325	0.227	4.408	
	*Ecohydrology*	Soil moisture storage abilities	−0.099	0.685	0.325	3.075	
		Catena influences	0.191	0.349	0.195	5.136	
		Type of plant community	0.140	0.594	0.385	2.600	
		Land degradation	0.463	0.016	0.223	4.479	

VIF, variance inflation factor; WRS, wet resource state; DRS, dry resource state.

a , Dependent variable: lnWRS.

b , Dependent variable: lnDRS.

Because our data showed slight skewness, we followed Brown and Forsythe’s (1974) suggestion, who argued that Levene’s test that uses the median performs best when the underlying data follow a skewed distribution. We, therefore, performed such a test for both the WRS (Levene’s statistic = 1.397; *df* = 23, 101; *p* = 0.131) and the DRS (Levene’s statistic = 2.236; *df* = 9, 42; *p* = 0.075). The test results were not significant (*p* > 0.05) in both resource states compelling us to reject the null hypothesis that equal variances are not assumed in the data set. The final regression model shown in Table 6 reveals a number of critical ecohydrological variables that are significantly shaping the amount of ESs that resource users are obtaining from their immediate landscapes.

Critical ecohydrological parameters under the WRS include flood characteristics (*p* < 0.001), catena influence (*p* < 0.1), type of plant community (*p* < 0.05) and land degradation (*p* < 0.01). We found such results to be concordant with the mainstream literature on ecohydrology and ESs. In the DRS, we however found only one significant ecohydrological variable. We also found a significant negative association between the amount of provisioning ESs collected by households and their relative income status (i.e. *β* = 0.543; *p* < 0.01). Unlike in the WRS where we find households occupying less degraded ecosystems harvesting more ES provisions (*β* = -2.55), we observe a significant and positive relationship between land degradation and the collection of provisioning ESs (i.e. *β* = 0.463; *p* < 0.05). Such results surprisingly implied that households who obtain provision ESs in degraded landscapes were obtaining more under the DRS as compared to those who occupied less degraded environments. Further analysis showed that this was because communities occupying degraded landscapes were faced with few alternative resource options forcing them to travel long distances transcending their own local boundaries to fetch ESs in distant commercial farms where supplies were relatively better.

## Conclusion

We noted that ecohydrological changes characterising the semi-arid landscapes of *Colophospermum mopane* are threatening the supply of provisioning ESs. In concordant with extant literature in the field, we observed four risk and/or threat management scenarios depicting different resource thresholds. The majority (77%) of the sampled households occupy two of such resource thresholds, where ES provisioning is either at the risk of being lost or is undersupplied to the extent that the livelihoods of households occupying such clusters are threatened. Critical ecohydrological parameters driving such risk management scenarios included flood characteristics, catena influence and land degradation for the WRS. For the DRS, we found land degradation to be the driving factor. The majority of households whose livelihoods are dependent on ES provisioning are at risk because they obtain ESs from downstream landscapes where the flood conditions do not permit soil moisture availability for plant and other ESs. This finding did not come as a surprise as volume decreases because of *transmission losses* resulting from infiltration of floodwaters are expected as one moves from upstream to downstream landscapes (Kelly & Olsen 1993; Tooth 2000). We also found out that such landscapes are largely degraded and that they occupy lower catena sections where vegetation cover, soil structure and hydrological conditions are least attractive and therefore supporting less ESs (Davies et al. 2014; Levick et al. 2010).

To safeguard the livelihoods of households depending on ES provisioning obtained from such landscapes, we have recommended a number of ecosystemic or conservation practices. To avoid further deterioration in soil quality and soil structure, we recommend gully filling. Most of the land degradation has been associated with the loss of valuable soil nutrients capable of supporting more ESs. Although deforestation is almost impossible to reverse in such environs, we recommend resource conservation practices such as pollarding and coppicing as such technical interventions will not result in the complete loss of vegetation. Sparse vegetation cover has offered such landscapes limited protection against further vegetation degradation, salinisation, soil compaction and nutrient loss (Pravalie 2016). Tree planting will not only curb land degradation but will go a long way towards ensuring that more ESs provisioning are available for future consumption (Buntinga et al. 2017; Li et al. 2014; Xu et al. 2015). We also underscore the need to streamline land degradation intervention according to known land degradation scenarios as recommended by UNCCD (2013). It is therefore essential that land under ‘non-degrading use’ and therefore requiring sustainable land use practices is identified. Similarly, land under ‘degrading use’ and therefore needing mitigation should be identified. Lastly, land already degraded and requiring restoration measures needs to be identified and appropriate action taken (Chasek et al. 2015; Grainger 2015).

Where agriculture is practised, we encourage communities to engage in conservation tillage as this will result in increased infiltration, as well as decreased evaporation – attributes that are so crucial to soil moisture availability. We also encourage greater participation of low-income households in the implementation of such measures, as they are not only the culprits of land degradation but also the most affected. Although high-income families may be contributing more to land degradation, they are affected less as we found them to be having alternative livelihood options at their disposal.
